# Micro-patterned deposition of MoS_2_ ultrathin-films by a controlled droplet dragging approach

**DOI:** 10.1038/s41598-021-93278-6

**Published:** 2021-07-07

**Authors:** Devendra Pareek, Kathryna G. Roach, Marco A. Gonzalez, Lukas Büsing, Jürgen Parisi, Levent Gütay, Sascha Schäfer

**Affiliations:** Ultrafast Nanoscale Dynamics, Institute of Physics, Carl Von Ossietzky University of Oldenburg, Oldenburg, Germany

**Keywords:** Materials science, Materials for devices, Nanoscale materials, Techniques and instrumentation, Chemical synthesis

## Abstract

Micropatterning of transition metal dichalcogenide (TMDC) ultrathin-films and monolayers has been demonstrated by various multi-step approaches. However, directly achieving a patterned growth of TMDC films is still considered to be challenging. Here, we report a solution-based approach for the synthesis of patterned MoS_2_ layers by dragging a precursor solution droplet with variable velocities across a substrate. Utilizing the pronounced shearing velocity dependence in a Landau-Levich deposition regime, MoS_2_ films with a spatially modulated thickness with alternating mono/bi- and few-layer regions are obtained after precursor annealing. Generally, the presented facile methodology allows for the direct preparation of micro-structured functional materials, extendable to other TMDC materials and even van der Waals heterostructures.

## Introduction

In the past decade, two-dimensional transition metal dichalcogenides (TMDCs), such as MoS_2_ or WS_2_, have been studied in detail and are now on the verge of being implemented in functional devices, ranging from highly efficient photo detectors^1,2^ and single photon emitters^3,4^ to lightweight and ultrathin flexible electronics^5,6^.

Popular vapor-based techniques for producing large-area MoS_2_ ultrathin films include chemical vapour deposition (CVD)^7^, and atomic layer deposition (ALD)^8^. For integrating TMDC films into functional devices and complex nano architectures, various patterning and interfacing approaches have been developed such as focused ion beam (FIB) milling^9^, photo and electron beam lithography^10^, and combinations of metal sputtering processes with selective etching after photolithographically defined masking^11^.

Despite the success and wide application of post-patterning approaches in current microelectronics, solution-based methods offer a unique set of handles to directly achieve a patterned growth, by applying techniques such as chemical inkjet printing^12,13^, nano stamping^14^, self-assembly^15^ or local electrochemical decomposition^16–18^.

Compared to CVD and ALD, the solution-based preparation of MoS_2_ films is much less studied. One line of research focuses on the deposition of colloidal MoS_2_ flakes and was successfully employed for the preparation of nano-porous MoS_2_ films, which act, for example, as a catalyst for the hydrogen evolution reaction^19–22^. Another promising solution-based approach starts with an inorganic precursor complex, containing thiomolybdate anions, subsequently decomposed into MoS_2_^23,24^. The decomposition reaction is usually performed by annealing the sample at elevated temperatures (> 500 °C) under an inert or H_2_/N_2_ atmosphere^23^. Such an approach appears to be suitable for producing large-scale and low cost thin-films of MoS_2_. For the deposition of the precursor solution, several techniques have been reported, including spin-^6,25–28^ and dip-coating^23,29^, which yielded homogeneous MoS_2_ layer formation with full sample coverage. Most studies report MoS_2_ bilayer, trilayer or thicker film formation^6,23,25–27,29^, with limited success in achieving MoS_2_ monolayers in small regions^28^. Patterned solution deposition approaches using thiomolybdate precursor solutions have not been reported so far.

Here, we present a novel solution-based droplet dragging approach for synthesizing patterned MoS_2_ thin-films. This wet–chemical method comprises the microscopically controlled meniscus guided deposition of a MoS_2_ precursor ink in a thin layer across a sapphire substrate, followed by a high-temperature reaction step for MoS_2_ formation. The obtained pattern consists of alternating mono-/bilayer and few-layer MoS_2_ regions, forming a microscale patterned TMDC film.

## Results and discussion

**Figure 1:**
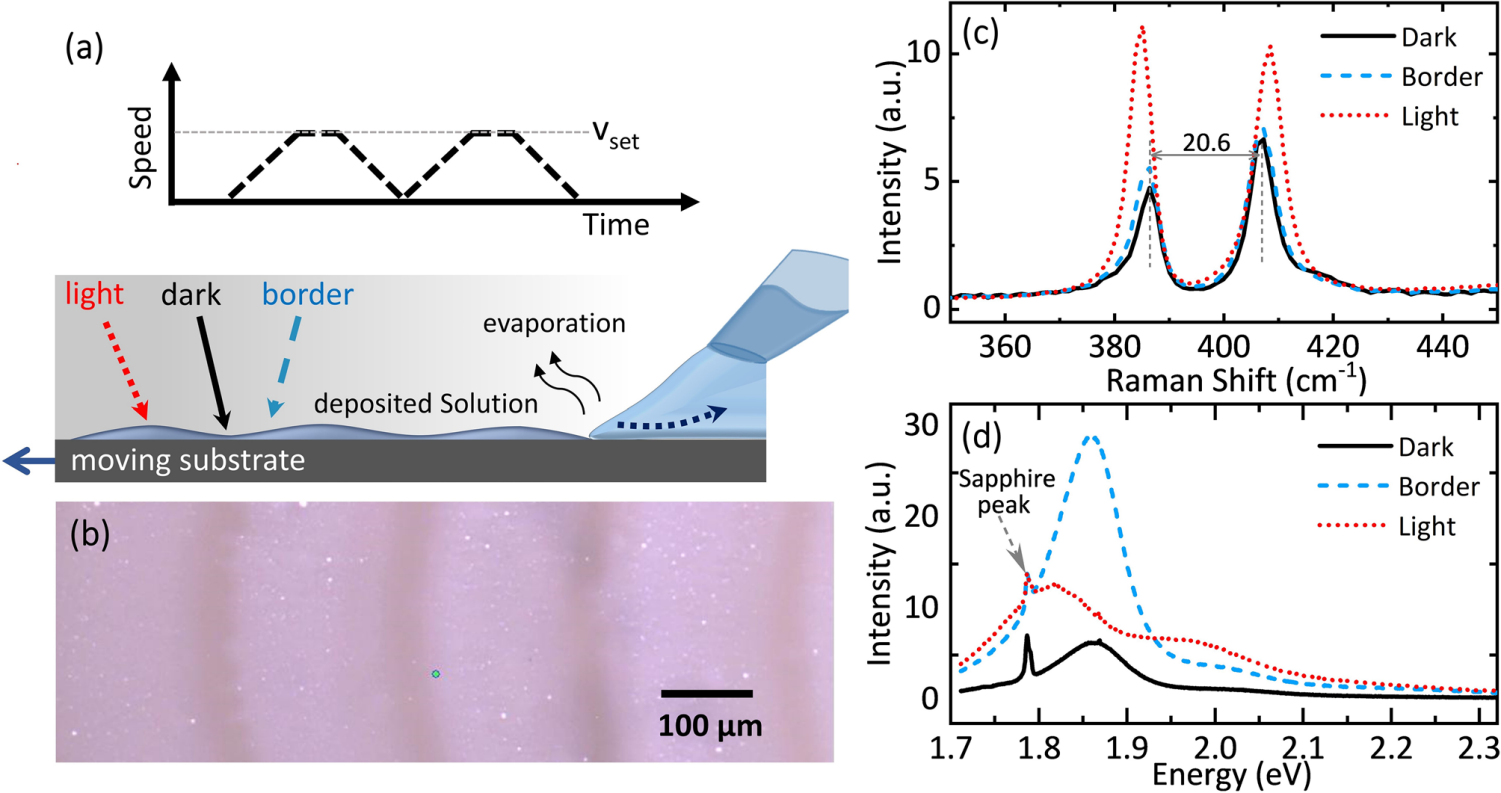
Patterned MoS_2_ deposition by a droplet dragging approach. (**a**) Schematic of the experimental set-up in which a precursor droplet is dragged across the substrate. Depending on the transient shape of the dragged droplet, a variation in the obtained precursor film thickness is obtained (bottom panel). Upper panel: Dragging speed. (**b**) Optical image of a patterned MoS_2_ film prepared by the droplet dragging approach (after annealing, droplet shearing velocity of 67 µm/s, with halts every 200 µm). (**c**,**d**) Representative Raman (**c**) and PL (**d**) spectra recorded in the dark, border and light MoS_2_ regions indicated in (**a**,**b**).

For the controlled deposition of the molybdenum precursor complex, we developed an approach, in which a precursor droplet ((NH_4_)_2_MoS_4_ in dimethyl-formamide (DMF)) suspended from the tip of a micropipette is dragged across a sapphire single-crystalline substrate by a controlled substrate movement (Fig. 1a, for details see “Experimental” section). Depending on the shearing velocity $v$ imparted by the moving substrate, the precursor droplet is deformed and leaves a thin trailing liquid film meniscus behind. Rapid evaporation from this meniscus results in the local deposition of precursor material. The wetting behaviour of the droplet can be adjusted by the choice of solvent, solvent additives, and the surface treatment of the substrate. In the present case, we found that a controlled layer formation is achieved by applying an initial oxygen plasma etching of the substrate and using DMF as a solvent. For spatially modulating the amount of local precursor deposition, we periodically halted the movement, so that the droplet intermittently adopts a sessile spherical shape with reduced solvent evaporation rate.

After annealing in a H_2_/N_2_ atmosphere at 900 °C, a distinct film deposition is visible on the substrate, as shown in Fig. 1b, with a spatial contrast periodicity corresponding to the distance between substrate halts (periodicity of 200 µm in the current case; for 100 µm periodicity see Supplementary Information, Fig. S1). The light and dark regions of the pattern correspond to MoS_2_ film regions with varying thickness. As shown below, the light regions (about 150–175 µm in width) are deposited from the trailing thin film during droplet movement, the darker regions (about 30–50 µm in width) are formed when the droplet shearing velocity is close to zero.

For the characterization of the MoS_2_ layer, we recorded micro-Raman and photoluminescence spectra on the various regions of the patterned samples. Representative Raman spectra on the light and dark region of the pattern are shown in Fig. 1c, exhibiting two characteristic peaks corresponding to MoS_2_ phonon modes involving the out-of-plane vibration of S atoms (A_1g_), at about 406 cm^−1^ to 410 cm^−1^, and the in-plane vibration of Mo and S atoms (E^1^_2g_), at 385 cm^−1^ to 386 cm^−1^. The observed distance *δ* between both peaks, ranging from 20 to 25 cm^−1^, serves as an indicator for the number of MoS_2_ layers in the film^27,30,31^.

In particular, within the dark region and up to the border to the light region, the peak spacing of 20.6 cm^−1^ and 20.9 cm^−1^, respectively, indicates the presence of MoS_2_ mono- to bilayer films. In contrast, the bright image regions show a larger Raman peak spacing of 23.5 cm^−1^, pointing to the formation of a few-layer (≤ 4 layers) film. Similarly, the photoluminescence (PL) spectra change significantly across the ripple structure, showing a narrow peak at 1.86 eV in the darker region and at the border between light and dark regions, and a broader double peaked spectrum (at 1.82 and 1.96 eV) in the lighter region (Fig. 1d). These observations are in line with previous work^32–36^ linking the change in PL spectra to the transition from an indirect to a direct semiconductor when decreasing the MoS_2_ thickness to a monolayer. Curiously, the PL spectra in the border region show the same spectral shape as in the darker region but with largely enhanced intensity.

**Figure 2:**
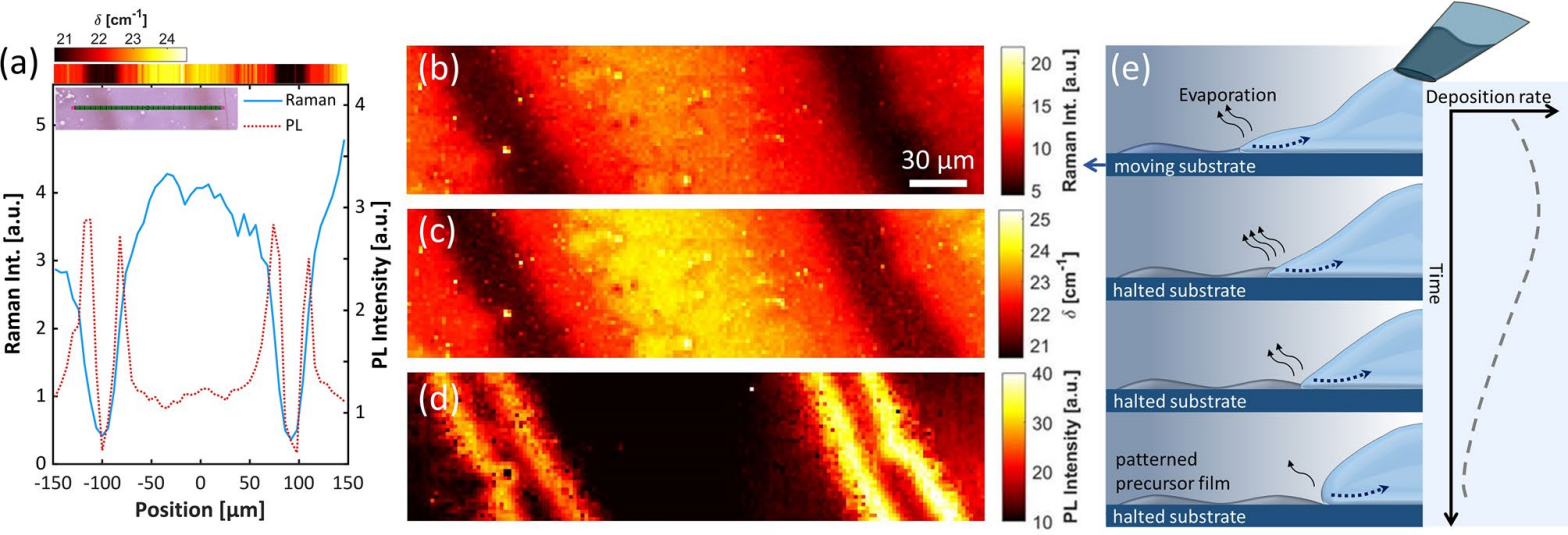
Spectroscopic MoS_2_ characterization and mechanism of spatially varying MoS_2_ deposition. (**a**) Raman and PL spectra mapped along a 300-µm line within the MoS_2_ ripple pattern (step size: 2 µm; microscopy image of the sample area shown in the inset; sample preparation parameters: droplet-dragging speed of 67 µm/s, halts every 200 µm). For each measurement position, the combined intensity of both Raman lines (blue curve) and the PL intensity (dashed, orange curve) are analysed. The extracted Raman peak-to-peak values δ are printed as a color coded band (top). All signals are obtained by averaging over spectra from three adjacent probing positions. (**b**–**d**) Raman and PL mapping of a 300 µm × 80 µm area (sample preparation parameters: droplet-dragging speed of 80 µm/s, halts every 200 µm). The darker regions in panel (**b**) and (**c**) indicate areas with monolayer domains with low coverage (small Raman intensity and low δ value). In panel (**d**), the highly luminescent domains related to monolayer-MoS_2_ are visible on the border regions. (**e**) Schematic representation of the shape of the dragged droplet and its change when halting the relative movement of the needle. The dynamics of the receding part of the trailing droplet results in a spatial modulation of the local precursor deposition.

For further quantification of the changing optical properties across the ripple pattern, we performed Raman and PL line scans (300-µm scanning length, 2-µm step size, optical focal spot size about 1 µm, Fig. 2a) and area mappings (300 × 80 µm^2^, 2-µm step size, Fig. 2b–d) and analysed at each point the overall PL intensity as well as the Raman intensity and peak position. Nonzero Raman and PL intensity across the whole pattern (Fig. 2b,d) indicates the presence of MoS_2_ material at each probing position. The large PL intensity observed in the border region coincides with Raman peak spacings of about 20 cm^−1^ (Fig. 2a top colour scale, Fig. 2c), signifying the formation of a MoS_2_ mono-/bilayer with high surface coverage. In the adjacent dark region, a similar Raman peak spacing and PL spectral shape as in the border region but with reduced PL and Raman intensity points to a mono-/bilayer film with low surface coverage. We note that depending on the overall amount of deposited material, for some samples we observed a gradual increase of PL towards the centre of the dark region (see Supplementary Information Fig. S1), indicating that in these cases a monolayer with uniformly high surface coverage was formed throughout this region.

**Figure 3:**
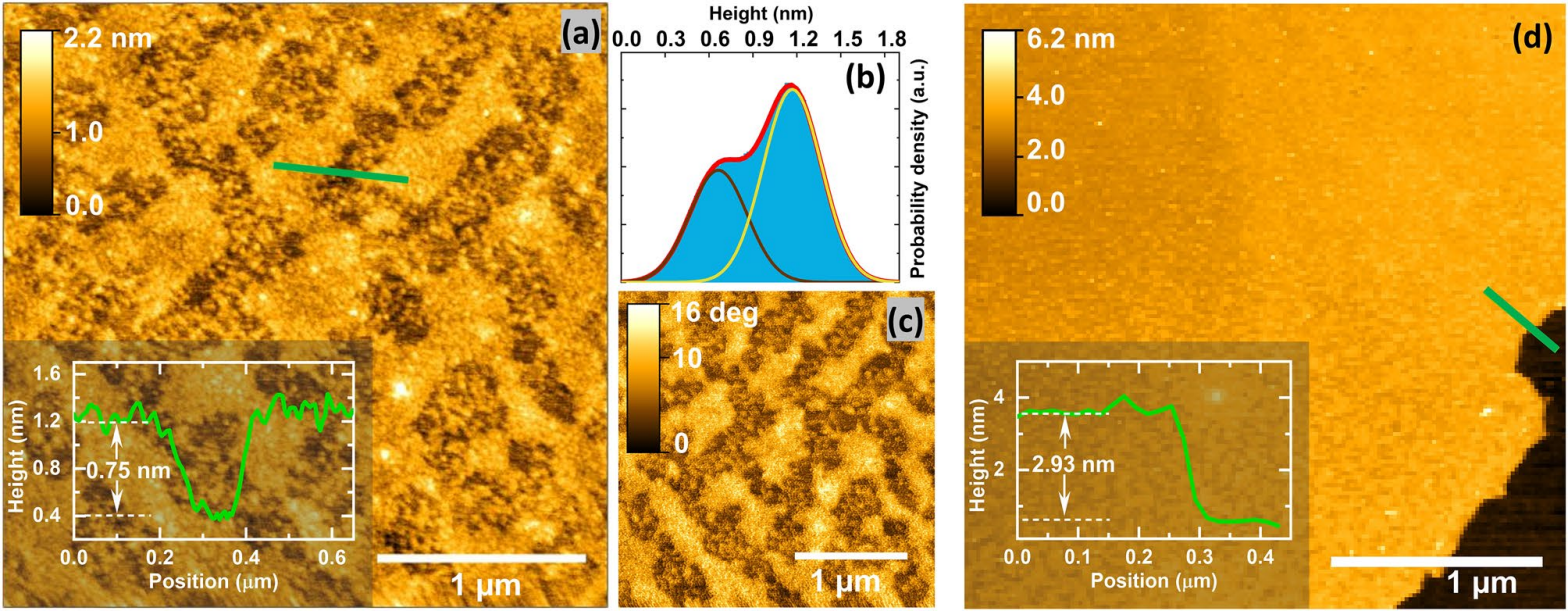
Atomic force micrographs of the annealed MoS_2_ thin films. (**a**,**c**) AFM topography (**a**) and phase (**c**) micrographs of an optically dark region of the sample shown in Fig. 1b. (**b**) Height distribution extracted from the image shown in (**a**). (**d**) Topographic AFM micrograph recorded at the centre of an optically bright region. Insets: Exemplary height cross-section transversely averaged over about 45 nm.

The inferred MoS_2_ layer thickness from the spectroscopic measurements was further corroborated by atomic force microscopy imaging (AFM) of the optically dark and bright regions of the sample shown in Fig. 1b. The AFM micrographs from the optically dark regions show in both the topographic (Fig. 3a) and the phase channel (Fig. 3c), elongated domains with a width of about 200–300 nm and a length of about 1 µm, which are separated by regions with dot-like 10-nm-sized features. The height histogram (Fig. 3b) is dominated by two peaks at about 0.6 and 1.2 nm, which might be related to adjacent monolayer and bilayer regions, or to a monolayer on a roughened support. In the centre of the optically bright regions, step heights in the range of 4–5 MoS_2_ layers are obtained, as shown in Fig. 3d.

To gain a mechanistic insight into the observed spatially varying material deposition, we consider the transient shape of the precursor droplet dragged across the substrate. As schematically indicated in Fig. 2e, a droplet dragged with a constant speed relative to the substrate adopts an asymmetric shape along the direction of the movement (upper panel), similar to some degree to a droplet under the influence of gravity on an inclined plane^37^.

Depending on the dragging velocity, the temperature and the surface tensions involved, different fluid-dynamical regimes exist for an evaporating dragged droplet. In the so-called Landau–Levich regime^38,39^, which is adopted for larger dragging speed, the droplet sheds a trailing thin film with a film thickness scaling with $C{a}^{2/3}$ (capillary number $Ca=\frac{\mu v}{\gamma }$; $\mu$: viscosity; $\gamma$: surface tension) and thus increasing with the shearing velocity ${v}^{2/3}$^38–40^. For an evaporating solution, efficient solvent loss, and thereby predominant solute deposition, occurs in this thin film section of the dragged droplet and the initial film thickness is a direct measure of the resulting solute thin film thickness. At low velocities, or at high evaporation rates (i.e. at high temperature and high solvent vapor pressure), no liquid thin film is trailing behind the droplet meniscus and evaporation occurs directly at the three-phase contact line. In this case, the so-called evaporative regime, the thickness of the deposited film scales inversely with the shearing velocity^39,40^, due to the corresponding decrease of the meniscus residence time above each part of the substrate. Therefore, at an intermediate velocity (i.e. in the transition region between both regimes) the thinnest films are deposited.

**Figure 4:**
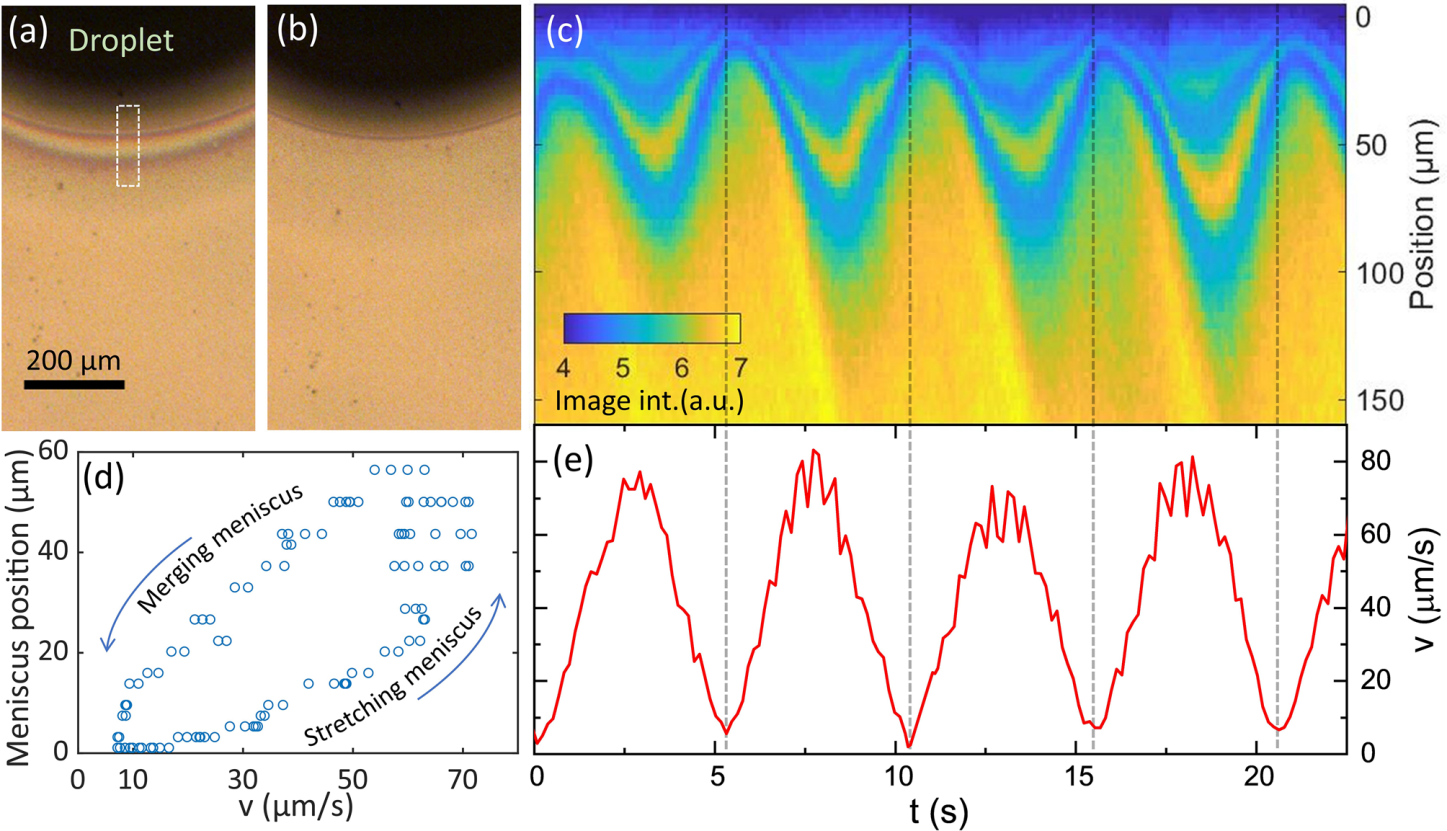
Transient meniscus shape and spatially modulated deposition (**a**,**b**) Optical micrograph of the precursor droplet (viewed from above) during different phases of the substrate movement (panel (**a**): t = 8.85 s and v = 48.6 µm/s; panel (**b**): t = 10.95 s and v = 19.5 µm/s). Interference fringes close to the droplet boundary indicated the thin region of the stretch liquid meniscus. (**c**) Intensity line profiles of the optical micrographs close to the meniscus recorded at different times. Temporal changes in the interference pattern of the meniscus and the local thin-film deposition density are visible. Line profiles correspond to the white-boxed area in panel (**a**) and are averaged along the horizontal direction. (**d**) Extracting from panel (**c**) the extend of the meniscus for different transient substrate speeds reveals that during speed cycling the meniscus does not adopt a steady-state geometry. (**e**) Extracted substrate velocity derived from the movement of local surface features. Slight differences with respect to the set-velocities (v_set_ = 67 µm/s) might be related to the inertia of the stage as well as small inaccuracies in the video frame analysis.

For observing the dynamically changed shearing velocity adopted in our experiments, the droplet shape was monitored from the top with a video camera (see Video S1 in the Supplementary Information). Exemplary video frames are shown in Fig. 4a,b for two phases of the substrate movement. Utilizing the movement of structural features on the substrate, we extracted the temporal evolution of the substrate movement, as shown in Fig. 4e.

The stretched droplet at a substrate velocity of 48.6 µm/s is visible in Fig. 4a by interference fringes close to the droplet boundary, corroborating that a Landau-Levich regime is reached. From the interference pattern, it can be deduced that the front region shows a thickness of below 105 nm, increasing to about 210 nm over a spatial distance of around 27 µm. For the substrate approximately at halt (Fig. 4b), the trailing thin film has merged with the main droplet and the liquid adopts the more symmetric shape of a sessile droplet (to some degree distorted by the presence of the deposition needle).

For further analysis, we extracted from each optical micrograph at different times a line profile close to the droplet boundary. As visible from Fig. 4c, the position of the meniscus boundary relative to the bulk of the droplet shows an oscillatory motion, in line with the schematics sketched in Fig. 2e. For the largest substrate velocities within one cycle the meniscus stretches about 60 µm further away from the droplet as compared to the case of the almost halted substrate. The length of the trailing thin film increases with the shearing velocity due to the increased initial liquid film thickness at constant evaporation rate. According to the scaling behaviour of Landau-Levich deposition, during these phases of movement, the thickest precursor films are expected to be deposited. For our case, we further corroborated the increased film thickness at larger velocities by adopting different peak shearing velocities and observing the difference in Raman peak spacing (see Supplementary Information Fig. S2). In related studies on meniscus-guided deposition, temporally modulated meniscus velocity or curvature were applied for patterned polymer deposition^41–43^.

For the phases of substrate movement with a small velocity (e.g. at around t = 10 s), we expect the deposition switching to the evaporative regime. However, due to the insufficient time of evaporation (in particular for the low vapor pressure of the solvent for our experimental conditions) and the diverging time to arrive at a steady-state condition in the evaporative regime, we do not expect a visible lateral growth of the film at low shearing velocities.

In addition, the spatio-temporal map in Fig. 4c directly indicates the movement phase, in which mono-/bilayer MoS_2_ is deposited. Due to different illumination conditions the bright regions visible in the as-deposited precursor layers in Fig. 4 corresponds to the dark-contrast mono/bi-layer regions of the resulting MoS_2_ layer after annealing as shown in Fig. 1b. The ultrathin regions are visible in Fig. 4c as diagonal streaks appearing at phases of smallest shearing velocity. Further evidence that ultrathin films are deposited at phases of close to zero shearing velocity is obtained by changing the spatial periodicity of the movement to 100 µm, resulting in a decreased width ratio of the multilayer region as compared to the mono-/bilayer region (see Supplementary Information Fig. S3).

Finally, we note that the specific shape of the moving droplet sensitively depends on the surface tension of the precursor solution and the surface energy of the substrate, critically influenced by the substrate pre-treatment and the choice of solvent or solvent additives. Specifically, we observed that for substrates exposed to oxygen plasma, for significantly longer than in the experiments presented before, the precursor droplet disconnects from the dragging needle and spontaneously spreads across the substrate. For non-plasma-treated substrates, the dragged droplet does not form an appreciably thin trailing film and no MoS_2_ deposition is observed.

In general, dragging the droplet with a varying velocity and acceleration across the substrate, even along nonlinear trajectories, gives access to different droplet shapes and thereby to a controlled local deposition from the trailing part of the droplet. Further experimental and theoretical studies are required to elucidate the detailed dependence of the droplet shape and deposition profile on the experimental parameters. In addition, it remains an open question to what extent the nanoscale morphology of the deposited precursor material is related to the structure of the MoS_2_ films after annealing, including its grain size, the amount of defects and type of grain boundaries.

Whereas in the current case a needle opening diameter of about 1 mm was utilized, microfluidic setups with micrometer scale tips may allow for a miniaturization of the demonstrated approach with deposition length scale in the few micrometer range. Furthermore, dragging a line-like liquid meniscus across the substrate surface may allow for patterned MoS_2_ deposition on large-area surfaces.

## Conclusion

In summary, we demonstrated a strategy for the controlled and directly patterned preparation of ultrathin MoS_2_ films without the need for post-structuring. The employed solution-based droplet-dragging approach results in films with alternating mono- and few-layer MoS_2_ regions with distinct optical properties. The quality of the uniformly patterned films may enable applications, such as the large-scale production of optical devices, including gratings or TMDC electrodes with an as-grown coplanar alignment.

## Experimental

Ammonium tetrathiomolybdate (ATM), (NH_4_)_2_MoS_4_, was dissolved in dimethylformamide (DMF) via sonication (@ 50 °C) to obtain a 2.6 wt.% precursor solution. The solution was filtered (0.45 µm pore diameter) and stored. Prior to use, the sapphire substrate surface was cleaned with isopropanol (IPA) and N_2_ gas. Afterwards, the substrate was plasma etched in an O_2_ atmosphere, to further remove surface contaminants and enhance the wettability of the surface^27^.

The as-synthesized ATM-DMF solution was spread on the cleaned sapphire substrate using a micropipette tip. For this purpose, we keep the position of the tip fixed and move the substrate using a motorised micromanipulator translation stage. The size of the protruding droplet from the tip was controlled by adjusting the liquid pressure using an attached syringe. Typical deposited precursor tracks showed a size of about 1.5 mm in width and up to 3 mm in length. Similar approaches were previously applied for direct self-assembly of nanoparticles^44^. For the reported experiments, we varied the maximum speed v_set_ of the substrate movement and included periodic halts of the sample, for varying the local deposition rate of the precursor material. For all cases, the acceleration was chosen so that v_set_ is achieved after 2 s.

After deposition, the samples were dried at 90 °C for 20 min and subsequently annealed (using an Annealsys AS-one 150 RTP system, 900 °C for 30 min in a H_2_/N_2_ (5%/95%) atmosphere) to form the MoS_2_ layer. The annealing process was terminated with a two-hour cooling period.

It was also observed that the spreading of the solution on the substrate surface was optimal for aged precursor solutions. Preliminary mass spectroscopy results indicate changes in the contained heteronuclear molybdenum complexes, presumably leading to changes in the surface chemistry of the precursor solution or a variation in the deposited precursor material. In addition, the formation of a trailing thin film depends on the individual surface tensions requiring a specific choice of solvent and substrate. For example, initial experiments using glass or SiO_2_/Si substrates and water or ethylene glycol as solvents did not result in a patterned deposition (for thermodynamic surface energies of DMF and sapphire, see Ref. 45–47). A video of the moving droplet on a sapphire substrate with and without plasma treatment is shown in the Supplementary Information Video S2.

Raman and Photoluminescence (PL) spectroscopy analyses of the samples were performed in a Horiba LabRAM Aramis confocal microscopy setup using a 457.9 nm excitation wavelength (spot size approximately 1 µm, excitation power of about 500 µW). The raw PL and Raman spectral data were fitted to extract peak intensities and positions. Raman A_1g_ and E^1^_2g_ peaks were each described by single Lorentzian functions with a common linear background. The PL emission peak was modeled by a superposition of three Lorentzian functions with a linear background.

## Supplementary Information

Supplementary Video captions.

Supplementary Figures 2.

Supplementary Video 1.

Supplementary Video 2.
