# Supplementary material for: Micro-patterned deposition of MoS2 ultrathin-films by a controlled droplet dragging approach
Source: Sci Rep. 2021 Jul 7;11:13993. doi: 10.1038/s41598-021-93278-6 (PMC8263556; doi:10.1038/s41598-021-93278-6)
Supplement: Supplementary file 1 — Supplementary Video captions. [file 41598_2021_93278_MOESM1_ESM.docx]

**Supplementary information: Video**

**Micro-patterned deposition of MoS_2_ ultrathin-films by a controlled droplet dragging approach**

Devendra Pareek*, Kathryna G. Roach*, Marco A. Gonzalez, Lukas Büsing, Jürgen Parisi,
Levent Gütay**, Sascha Schäfer**

Ultrafast Nanoscale Dynamics, Institute of Physics,
Carl von Ossietzky University of Oldenburg, Oldenburg, Germany

*) equal contributions

**) corresponding authors

**Supplementary video caption (Video-1):** Oscillating droplet meniscus during dynamically changed shearing velocity. Analysis of transient meniscus position during the deposition of the patterned film corresponding to Fig. 1a (droplet shearing velocity of 67 µm/s, with halts every 200 µm). Top left: Camara position during the recording of meniscus movement (top-right). Bottom panel: Line profiles correspond to the red-boxed area in the top-right panel, averaged along the horizontal direction, and plotted with time.

**Supplementary video caption (Video-2): Optical micrograph of the dragged precursor droplet on a sapphire substrate** with and without previous plasma treatment. Only in the plasma-treated substrate, a trailing liquid thin-film is observed. Camara position is same as in Video-1.
