# Supplementary material for: Micro-patterned deposition of MoS2 ultrathin-films by a controlled droplet dragging approach
Source: Sci Rep. 2021 Jul 7;11:13993. doi: 10.1038/s41598-021-93278-6 (PMC8263556; doi:10.1038/s41598-021-93278-6)
Supplement: Supplementary file 2 — Supplementary Figures 2. [file 41598_2021_93278_MOESM2_ESM.docx]

**Supplementary information**

**Micro-patterned deposition of MoS_2_ ultrathin-films by a controlled droplet dragging approach**

Devendra Pareek*, Kathryna G. Roach*, Marco A. Gonzalez, Lukas Büsing, Jürgen Parisi,
Levent Gütay**, Sascha Schäfer**

Ultrafast Nanoscale Dynamics, Institute of Physics,
Carl von Ossietzky University of Oldenburg, Oldenburg, Germany

*) equal contributions

**) corresponding authors

**
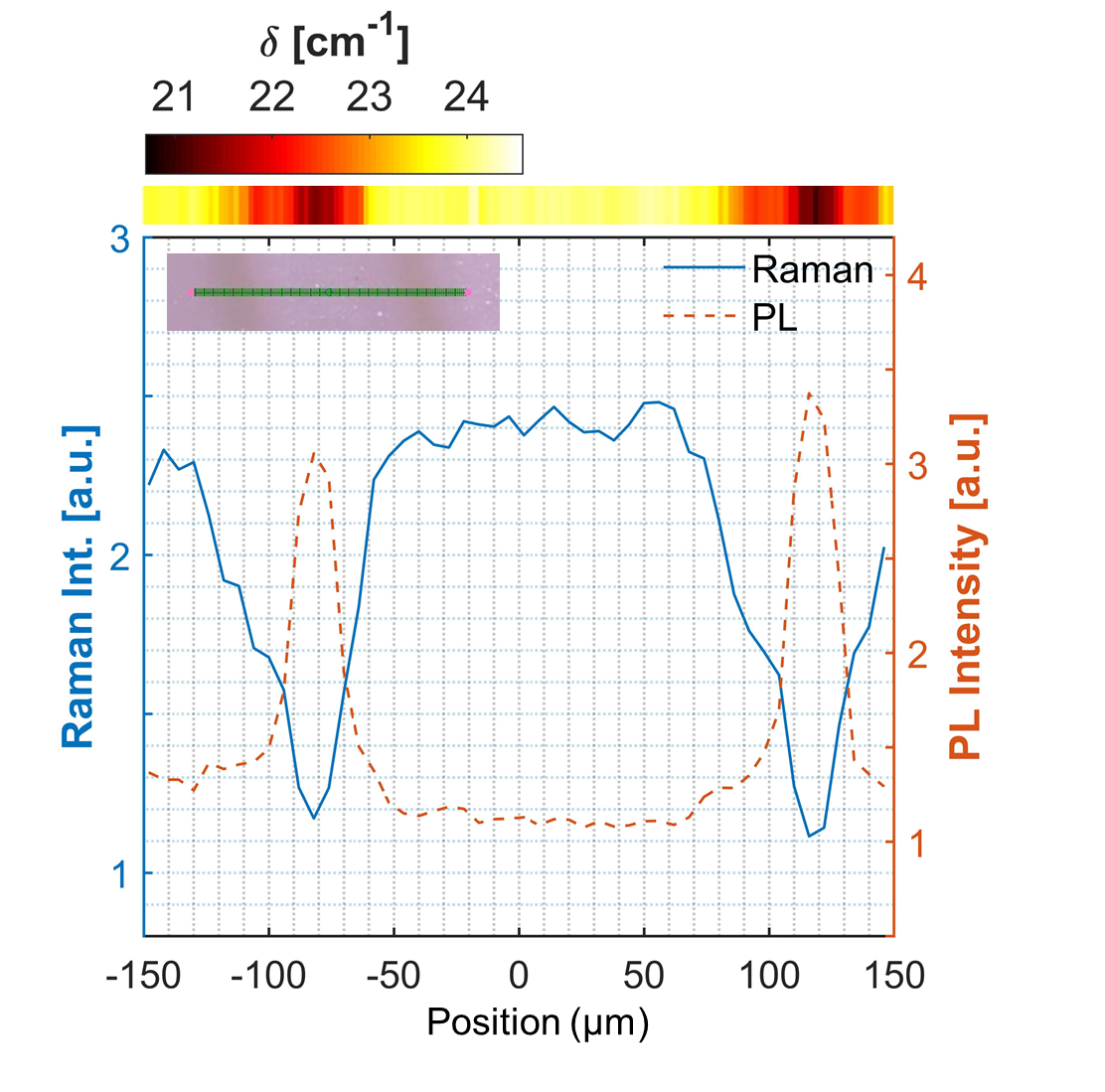
**

**Figure S1:** Raman and PL spectra line mapped along a 300-µm line within the MoS_2_ ripple pattern (step size: 2 µm; microscopy image of the sample area shown in the inset). A gradual increase of PL intensity towards the centre of the dark region indicating that in this case a monolayer with uniformly high surface coverage was formed throughout the dark region.

**Figure S2:** Raman spectra measured on the thick regions (on the locations similar to the light regions as shown in Fig.1) of the samples prepared at different shearing velocities (for details on velocity profiles, see main text). Larger peak separation (i.e. larger numbers of MoS_2_ layers) is corresponding to higher shearing velocities.

**
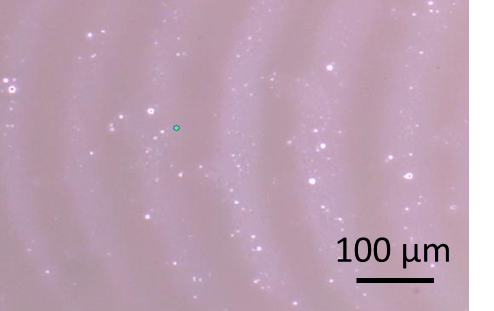
**

**Figure S3:** Optical image of a patterned MoS_2_ film prepared by the droplet dragging approach (after annealing, periodic halts of the substrate: every 100 µm).
